# Supplementary material for: Reconstructing the phylogeny and evolutionary history of freshwater fishes (Nemacheilidae) across Eurasia since early Eocene
Source: eLife. 2025 Apr 4;13:RP101080. doi: 10.7554/eLife.101080 (PMC11970906; doi:10.7554/eLife.101080)
Supplement: Supplementary file 1. — Voucher numbers starting with ‘A’ refer to the collection of IAPG, Liběchov, Czech Republic; ‘CMK’ numbers refer to the collection of Maurice Kottelat; ‘GenBank’ refers to sequences from GenBank; ‘ZRC’ to samples housed in the Lee Kong Chian Natural History Museum, National University of Singapore, Singapore. [file elife-101080-supp1.docx]

**Table S1.**

List of analysed samples, their identification, geographical origin, voucher number and GenBank accession numbers for their sequences. Voucher numbers starting with ‘A’ refer to the collection of IAPG, Liběchov, Czech Republic; ‘CMK’ numbers refer to the collection of Maurice Kottelat; ‘GenBank’ refers to sequences from GenBank; ‘ZRC’ to samples housed in the Lee Kong Chian Natural History Museum, National University of Singapore, Singapore.

| Species name | Country | Province | River drainage | voucher | Cyt b | RAG 1 | IRBP2 | MYH6 | RH 1 | EGR 3 |
| --- | --- | --- | --- | --- | --- | --- | --- | --- | --- | --- |
|  |  |  |  |  |  |  |  |  |  |  |
| EASTERN CLADE | |  |  |  |  |  |  |  |  |  |
| *Karstsinnectes acridorsalis* | China | Guangxi | Pearl | GenBank | ON116515 | OP473644 | - | OP473686 | OP473776 | OP473850 |
| *Karstsinnectes anophthalmus* | China | Guangxi | Pearl | GenBank | ON116506 | OP473637 | OP473907 | OP473664 | OP473763 | OP473828 |
| *Karstsinnectes parvus* | China | Guangxi | Pearl | GenBank | ON116520 | OP473651 | OP473900 | OP473693 | - | OP473859 |
| *Lefua costata* | Korea | Gangwon | Cheon Jin Cheon | A1895  A6942 | PP279919  KP738591 | PP315753  KP738551 | PP280130  KP738511 | PP280341  OL191348 | -  - | -  PP259693 |
| *Lefua torrentis* | Japan | Hyogo | Kako | A1050  A1051  A1052 | PP279878  PP279879  PP279880 | PP315711  PP315712  PP315714 | PP280091  PP280092  PP280093 | PP280288  PP280289  PP280291 | PP259745  PP259747  PP259748 | -  -  - |
| *Micronemacheilus bailianensis* | China | Guangxi | Pearl | GenBank | ON116504 | OP473620 | OP473876 | OP473662 | OP473759 | OP473825 |
| *Micronemacheilus cruciatus* | Vietnam | Thua Tien-Hue | Song Bu Lu | A3293  A3294 | PP279965  PP279966 | PP315801  PP315802 | -  - | PP280394  PP280395 | PP259847  PP259848 | PP259670  PP259671 |
| *Micronemacheilus longibarbatus* | China | Guangxi | Pearl | GenBank | ON116508 | OP473625 | OP473879 | OP473666 | OP473761 | OP473833 |
| *Micronemacheilus pulcherimus* | China | Guangxi | Pearl | A8690  A8691 | PP280043  PP280044 | PP315878  PP315879 | PP280229  PP280230 | PP280508  PP280509 | PP259968  PP259969 | PP259700  - |
| *Oreonectes guananensis* | China | Guangxi | Pearl | GenBank | ON116507 | OP473623 | OP473878 | OP473665 | OP473750 | OP473830 |
| *Oreonectes luochengensis* | China | Guangxi | Pearl | GenBank | ON116495 | - | OP473882 | OP473670 | OP473749 | OP473836 |
| *Oreonectes platycephalus* | China | Hong Kong | Tai Tam | A1674  A1675  A1676  GenBank | PP279902  PP279903  PP279904  ON116528 | PP315737  PP315738  PP315739  OP473652 | PP280115  PP280116  PP280117  OP473904 | PP280323  PP280324  PP280325  OP473696 | PP259777  PP259778  PP259779  OP473745 | -  -  -  OP473862 |
| *Oreonectes* cf. *platycephalus 1* | China | Guangxi | Pearl | A9065 | PP280053 | PP315888 | PP280239 | PP280518 | PP259977 | PP259707 |
| *Oreonectes* cf. *platycephalus 2* | China | Guangxi | Pearl | A8697 | PP280045 | PP315880 | PP280231 | PP280510 | PP259970 | PP259701 |
| *Oreonectes* cf. *platycephalus 3* | Vietnam | Lang Son | Pearl | A10603 | PP279881 | - | - | PP280292 | - | - |
| *Oreonectes polystigmus* | China | Guangxi | Pearl | GenBank | ON116514 | OP473614 | OP473871 | OP473657 | OP473746 | OP473856 |
| *Oreonectes* sp. | China | Guangxi | Pearl | A8963  A8964 | PP280047  PP280048 | PP315882  PP315883 | PP280233  PP280234 | PP280512  PP280513 | PP259972  - | PP259702  PP259703 |
| *Paranemachilus genilepis* | China | Guangxi | Pearl | GenBank | ON116497 | OP473630 | OP473885 | OP473673 | OP473752 | OP473839 |
| *Paranemachilus pingguoensis* | China | Guangxi | Pearl | GenBank | ON116500 | OP473634 | OP473888 | OP473676 | OP473755 | OP473843 |
| *Paranemachilus zhengbaoshani* | China | Guangxi | Pearl | A9153 | PP280054 | PP315889 | PP280240 | PP280519 | PP259978 | PP259708 |
| *Sundoreonectes sabanus* | Malaysia | Sabah | Baram | A1844  A1845 | PP279914  PP279915 | PP315748  PP315749 | PP280126  PP280127 | PP280334  PP280335 | PP259789  PP259790 | PP259651  PP259652 |
| *Traccatichthys pulcher* | China | Guangxi | Pearl | A1804  A1805  A8681 | PP279910  PP279911  PP280042 | PP315744  PP315745  PP315877 | PP280122  PP280123  PP280228 | PP280330  PP280331  PP280507 | PP259785  PP259786  PP259967 | -  -  PP259699 |
| *Traccatichthys taeniatus* | Vietnam  Laos | Nghe An  Houaphan | Lam  Lam | A3175  A3176  CMK 25931 | PP279960  PP279961  PP279871 | PP315796  PP315797  PP315710 | PP280167  PP280168  PP280088 | PP280387  PP280388  PP280249 | PP259840  PP259841  PP259738 | PP259666  -  - |
| *Traccatichthys* cf. *taeniatus* | Vietnam | Quang Nam | Cau Do | A9575 | PP280060 | - | - | - | - | - |
| *Traccatichthys zispi* | China | Hainan | - | GenBank | ON116518 | OP473648 | OP473898 | OP473691 | OP473780 | OP473861 |
| *Troglonectes barbatus* | China | Guizhou | Pearl | GenBank | ON116501 | OP473635 | OP473889 | OP473678 | OP473756 | - |
| *Troglonectes daqikongensis* | China | Guizhou | Pearl | GenBank | ON116526 | OP473641 | - | OP473683 | OP473773 | OP473849 |
| *Troglonectes dongganensis* | China | Guangxi | Pearl | GenBank | ON116503 | OP473617 | OP473875 | OP473661 | OP473757 | OP473847 |
| *Troglonectes donglanensis* | China | Guangxi | Pearl | GenBank | ON116505 | OP473621 | OP473877 | OP473663 | OP473762 | OP473826 |
| *Troglonectes duanensis* | China | Guangxi | Pearl | GenBank | ON116509 | OP473622 | OP473880 | OP473667 | OP473764 | OP473831 |
| *Troglonectes elongatus* | China | Guangxi | Pearl | GenBank | ON116502 | OP473616 | OP473874 | OP473660 | OP473758 | OP473848 |
| *Troglonectes furcocaudalis* | China | Guangxi | Pearl | GenBank | ON116512 | OP473628 | OP473883 | OP473671 | OP473767 | OP473837 |
| *Troglonectes jiarongensis* | China | Guizhou | Pearl | GenBank | ON116527 | OP473643 | OP473894 | OP473685 | OP473769 | OP473846 |
| *Troglonectes lihuensis* | China | Guangxi | Pearl | GenBank | ON148332 | OP473618 | OP473892 | OP473688 | - | OP473852 |
| *Troglonectes macrolepis* | China | Guangxi | Pearl | GenBank | ON116498 | OP473632 | OP473886 | OP473674 | OP473753 | OP473841 |
| *Troglonectes microphthalmus* | China | Guangxi | Pearl | A8988  A8989  GenBank | PP280049  PP280050  ON116494 | PP315884  PP315885  OP473631 | PP280235  PP280236  OP473872 | PP280514  PP280515  OP473659 | PP259973  PP259974  OP473748 | PP259704  PP259705  OP473840 |
| *Troglonectes retrodorsalis* | China | Guangxi | Pearl | GenBank | ON116511 | OP473627 | OP473873 | OP473669 | OP473766 | OP473835 |
| *Troglonectes shuilongensis* | China | Guizhou | Pearl | GenBank | ON116522 | OP473636 | OP473891 | OP473679 | OP473768 | OP473834 |
| *Troglonectes translucens* | China | Guangxi | Pearl | GenBank | ON116510 | OP473626 | OP473881 | OP473668 | OP473765 | OP473832 |
| *Yunnanilus pleurotaenia* | China | Yunnan | Yangtze | A2967  A2968  A2969 | PP279954  PP279955  PP279956 | PP315791  PP315792  PP315793 | PP280163  PP280164  PP280165 | PP280381  PP280382  PP280383 | PP259836  PP259837  PP259838 | PP259664  PP259665  - |
|  |  |  |  |  |  |  |  |  |  |  |
| NORTHERN CLADE | |  |  |  |  |  |  |  |  |  |
| *Barbatula barbatula* | France  Russia  Germany  Czechia  Czechia  Poland | Haute-Garonne  Unknown  Nordrhein-Westfalen  Liberecky  Stredocesky  Dolnośląskie | Garonne  unknown  Rhine  Elbe  Elbe  Oder | CMK18464_1CMK18464_2  A4013  A4015  A2046  A2047  A2587  A2588  A8393  A8394  A2957  A2958 | PP279842  PP279843  PP279979  PP279980  PP279922  PP279923  PP279923  PP279941  KP738604  KP738605  PP279952  PP279953 | PP315677  PP315678  PP315816  PP315817  PP315756  PP315757  PP315777  PP315778  KP738564  KP738565  PP315789  PP315790 | -  -  PP280179  PP280180  PP280133  PP280134  PP280150  PP280151  KP738524  KP738525  PP280161  PP280162 | PP280251  PP280252  PP280409  PP280410  PP280344  PP280345  PP280367  PP280368  PP280499  OL191359  PP280379  PP280380 | -  PP259711  PP259858  -  PP259798  PP259799  PP259822  PP259823  PP259955  PP259956  PP259834  PP259835 | -  PP259624  -  PP259674  PP259655  -  -  -  PP259697  -  -  - |
| *Barbatula* cf. *compressirostris* | Mongolia  Mongolia | Khovd  Khovd | Khovd Tsendkhar  Khovd | CMK19564_1 CMK19564_2  CMK19590_1CMK19590_2 | PP279848  PP279849  PP279852  PP279853 | PP315683  PP315684  PP315687  PP315688 | PP280067  PP280068  PP280071  PP280072 | PP280257  PP280258  PP280261  PP280262 | PP259716  PP259717  PP259720  PP259721 | PP259626  -  PP259628  - |
| *Barbatula dgebuadzei* | Mongolia | Bayankhongor | Baydrag | CMK19594_1  CMK19594_2 | PP279854  PP279855 | PP315689  PP315690 | PP280073  PP280074 | PP280263  PP280264 | PP259722  PP259723 | PP259629  - |
| *Barbatula karabanowi* | Mongolia | Khovd | Bulgan Gol | CMK19584_1 CMK19584_2 | PP279850  PP279851 | PP315685  PP315686 | PP280069  PP280070 | PP280259  PP280260 | PP259718  PP259719 | PP259627  - |
| *Barbatula oreas* | Japan | Hokkaido | Shinkawa | A11223  A11224 | PP279887  PP279888 | PP315722  PP315723 | PP280097  PP280098 | PP280300  PP280301 | PP259754  PP259755 | PP259638  - |
| *Barbatula* sp. Korea | Korea | Gangwon | Cheon Jin Cheon | A1888 | PP279918 | PP315752 | - | PP280340 | - | - |
| *Barbatula* sp. Tuul | Mongolia | Ulaanbaatar | Selenga | A4246  A4247  A4248  A4250 | PP279981  PP279982  PP279983  PP279985 | -  PP315818  PP315819  PP315821 | -  PP280181  PP280182  PP280184 | PP280412  PP280413  PP280414  PP280416 | PP259861  -  PP259862  PP259863 | PP259676  -  -  - |
| *Barbatula toni* | Russia | Primorsky | Amur | A3171 | PP279959 | - | - | PP280386 | - | - |
| *Barbatula* cf. *toni* | Mongolia  Mongolia | Khövgöl  Ulaanbaatar | Selenga  Selenga | CMK19540_1  CMK19540_2A4249 | PP279844  PP279845  PP279984 | PP315679  PP315680  PP315820 | PP280063  PP280064  PP280183 | PP280253  PP280254  PP280415 | PP259712  PP259713  - | -  -  PP259677 |
| *Claea dabryi* | China | Sichuan | Yangtze | GenBank | MG238214 | MG237922 | MG238312 | - | MG238015 | - |
| *Triplophysa baotianensis* | China | Guizhou | Pearl | GenBank | MT992550 | OP473612 | OP473868 | OP473655 | - | OP473864 |
| *Triplophysa bleekeri* | China | no detail | Yangtze | GenBank | MG238298  KX373847 | MG238003  MG725561 | MG238415  MG698830 | -  MG698529 | -  MG697779 | -  - |
| *Triplophysa brevicauda* | China | no detail | Yangtze | GenBank | MG238300 | MG238005 | MG238417 | - | MG238107 | - |
| *Triplophysa dalaica* | China | Gansu | no details | GenBank | MG697586 | - | MG698831 | MG698530 | MG697806 | - |
| *Triplophysa dorsalis* | Kazachstan | Almaty | Lake Balkash | A5377  A5378  A5380  A5383 | PP280001  PP280002  PP280004  PP280007 | PP315838  PP315839  PP315841  PP315844 | PP280191  PP280192  PP280194  PP280196 | PP280444  PP280445  PP280447  PP280450 | PP259893  PP259894  PP259895  PP259898 | PP259684  PP259685  -  - |
| *Triplophysa grahami* | China | Yunnan | Yangtze | A1663  A2933 | MK608125  PP279948 | OL191414  PP315785 | MT536722  - | OL191277  PP280375 | PP259776  PP259830 | PP259648  - |
| *Triplophysa gundriseri* | Mongolia | Khövgöl | Tes Gol | CMK19543_1  CMK19543_2 | PP279846  PP279847 | PP315681  PP315682 | PP280065  PP280066 | PP280255  PP280256 | PP259714  PP259715 | PP259625  - |
| *Triplophysa huapingensis* | China | Guangxi | Pearl | GenBank | MG697589 | - | MG698834 | MG698537 | MG697870 | - |
| *Trplophysa labiata* | Kazachstan | Almaty | Lake Balkash | A5381  A5384  A5385 | PP280005  PP280008  PP280009 | PP315842  PP315845  PP315846 | -  PP280197  PP280198 | PP280448  PP280451  PP280452 | PP259896  PP259899  PP259900 | -  -  - |
| *Triplophysa leptosoma* | China | no detail | Yangtze | GenBank | KX373839 | - | MG698825 | MG698524 | MG697601 | - |
| *Triplophysa luochengensis* | China | Guangxi | Pearl | A8990  A8991 | PP280051  PP280052 | PP315886  PP315887 | PP280237  PP280238 | PP280516  PP280517 | PP259975  PP259976 | PP259706  - |
| *Triplophysa nandanensis* | China | Guangxi | Pearl | GenBank | MG697588 | - | MG698833 | MG698536 | MG697869 | - |
| *Triplophysa nanpanjiangensis* | China | Yunnan | Pearl | GenBank | MG238302 | MG238007 | MG238419 | - | MG238109 | - |
| *Triplophysa nasobarbatula* | China | Guizhou | Pearl | GenBank | ON116529 | OP473653 | OP473869 | OP473697 | OP473781 | OP473865 |
| *Triplophysa obscura* | China | no detail | Yangtze | GenBank | MG238304 | MG238009 | MG238421 | - | MG238111 | - |
| *Triplophysa orientalis* | China | Qinghai | Yangtze | GenBank | KX373846 | - | MG698829 | MG698528 | MG697755 | - |
| *Triplophysa pseudoscleroptera* | China | Qinghai | Yangtze | GenBank | MG697585 | - | MG698828 | MG698527 | MG697684 | - |
| *Triplophysa rosa* | China | Wulong | Yangtze | GenBank | MG697587 | MG725565 | MG698832 | MG698535 | MG697868 | - |
| *Triplophysa scleroptera* | China | No details | Yangtze | GenBank | MG238307  KX373833 | MG238012  MG725554 | MG238424  MG698838 | MG698534 | MG238113  MG697908 | -  - |
| *Triplophysa siluroides* | China | no details | no details | 1797 | MT536720 | EF063156 | MT536723 | OL191278 | PP259784 | PP259650 |
| *Triplophysa stenura* | China | Yunnan | Yangtze | 2935  2938  2939  GenBank | PP279949  PP279950  PP279951  MG697583 | PP315786  PP315787  PP315788  MG725550 | PP280158  PP280159  PP280160  MG698824 | PP280376  PP280377  PP280378  MG698523 | PP259831  PP259832  PP259833  MG697592 | -  PP259662  PP259663  - |
| *Triplophysa stolickai* | China | no detail | no details | GenBank | MG697582 | MG725535 | MG698822 | MG698521 | MG697590 | - |
| *Triplophysa strauchi* | Kazachstan  Kyrgyzstan | Almaty  Naryn | Lake Balkash  Syr Darya | 5367  5379  5382  11496  11497 | PP280000  PP280003  PP280006  MT536721  PP279889 | -  PP315840  PP315843  OL191500  PP315725 | -  PP280193  PP280195  MT536724  PP280100 | -  PP280446  PP280449  OL191382  PP280303 | -  -  PP259897  PP259757  PP259758 | -  PP259686  -  PP259639  - |
| *Triplophysa tenuis* | China | no detail | no details | GenBank | MG697584 | MG725556 | MG698827 | MG698526 | MG697626 | - |
| *Triplophysa wuweiensis* | China | Gansu | no details | GenBank | KX373838 | - | MG698823 | MG698522 | MG697591 | - |
|  |  |  |  |  |  |  |  |  |  |  |
| INDOCHINESE CLADE | |  |  |  |  |  |  |  |  |  |
| *Homatula anguillioides* | China | Yunnan | Mekong | GenBank | HM010583 | HM010669 | MG238315 | - | MG238018 | - |
| *Homatula change* | China | Yunnan | Mekong | GenBank | - | - | MG238318 | - | MG238021 | - |
| *Homatula cryptoclathrata* | China | Yunnan | Salween | GenBank | HM010569 | HM010663 | MG238332 | - | - | - |
| *Homatula disparizona* | China | Yunnan | Red | GenBank | MG238218 | MG237926 | MG238321 | - | MG238023 | - |
| *Homatula laxiclathra* | China | Shanxi | Yellow | GenBank | MG238219 | MG237927 | MG238322 | - | MG238024 | - |
| *Homatula longidorsalis* | China | Yunnan | Pearl | GenBank | HM010522 | HM010618 | MG238324 | - | MG238026 | - |
| *Homatula potanini* | China | Sichuan | Yangtze | A1788  A1789 | PP279908  PP279909 | PP315742  PP315743 | PP280120  PP280121 | PP280328  PP280329 | PP259782  PP259783 | PP259649  - |
| *Homatula pycnolepis* | China | Yunnan | Mekong | A2973 | PP279957 | PP315794 | PP280166 | PP280384 | PP259839 | - |
| *Homatula variegata* | China | Sichuan | Yangtze | A1459  A1460 | PP279900  PP279901 | PP315735  PP315736 | PP280112  PP280113 | PP280319  PP280320 | PP259773  PP259774 | PP259646  - |
| *Homatula wuliangensis* | China | Yunnan | Mekong | GenBank | HM010517 | HM010609 | MG238336 | - | MG238036 | - |
| *‘Nemacheilus' arenicolus* | Laos | Bolikhamsay | Mekong | CMK21208_2  CMK21208_3 | MW512964  MW512965 | MW513090  MW513091 | MW513213  MW513214 | PP280265  PP280266 | PP259724  PP259725 | -  PP259630 |
| *‘Nemacheilus' argyrogaster* | Laos | Sekong | Mekong | CMK21521_1  CMK21521_2 | MW512988  MW512989 | MW513111  MW513112 | MW513237  MW513238 | PP280267  PP280268 | PP259726  PP259727 | PP259631  - |
| *‘Nemacheilus' banar* | Vietnam | Kontum | Mekong | A3307  A3308 | PP279967  MW512968 | PP315803  MW513094 | PP280172MW513217 | PP280396  PP280397 | PP259849  PP259850 | PP259672  - |
| *‘Nemacheilus' cleopatra* | Vietnam | Gia Lai | Song Ba | A3256  A3257 | MW512982  MW512983 | MW513106  MW513107 | MW513231  MW513232 | PP280392  PP280393 | PP259845  PP259846 | PP259669  - |
| *Rhyacoschistura suber* | Laos  Laos | Xiengkhuang  Xaysomboon | Mekong  Mekong | CMK22643  CMK22484 | PP279857  PP279856 | PP315692  PP315691 | -  PP280079 | PP280270  PP280269 | PP259729  PP259728 | PP259632  - |
| *Schistura amplizona* | China | Yunnan | Mekong | GenBank | MG238243 | MG237949 | MG238357 | - | MG238056 | - |
| *Schistura bolavensis* | Laos | Champasak | Mekong | A4618  A4620 | KP738575  KP738576 | KP738535  KP738536 | KP738495  KP738496 | OL191315  PP280421 | PP259869  PP259870 | PP259681  - |
| *Schistura bucculenta* | China | Yunnan | Mekong | GenBank | JN837654 | JN837666 | - | - | - | - |
| *Schistura callichroma* | China | Yunnan | Red | GenBank | MG238244 | MG237950 | MG238359 | - | MG238057 | - |
| *Schistura caudofurca* | China | Yunnan | Red | GenBank | MG238245 | MG237951 | MG238360 | - | MG238059 | - |
| *Schistura* cf. *amplizona* | Thailand | Loei | Mekong | A2375 | PP279932 | PP315766 | PP280144 | PP280355 | PP259808 | PP259658 |
| *Schistura* cf. *palma* | Thailand | Loei | Mekong | 2376 | PP279933 | PP315767 | - | PP280356 | PP259809 | PP259659 |
| *Schistura* cf. *schultzei* | Thailand | Loei | Mekong | 2522  2523 | PP279934  PP279935 | PP315768  PP315769 | PP280145  PP280146 | PP280359  PP280360 | PP259813  PP259814 | PP259660  - |
| *Schistura cryptofasciata* | China | Yunnan | Salween | GenBank | MG238250 | MG237956 | MG238366 | - | MG238063 | - |
| *Schistura desmotes* | Thailand | Chiang Mai | Chao Phraya | A1180  A1181  A1182 | PP279892  PP279893  PP279894 | PP315728  PP315729  PP315730 | -  -  - | PP280306  PP280307  PP280308 | PP259761  PP259762  PP259763 | PP259640  -  - |
| *Schistura dubia* | Thailand | Phrae | Chao Phraya | GenBank | MK301364 | - | - | - | - | - |
| *Schistura fasciolata* | China | Guangxi | Pearl | A5300  A5301  A5302 | KP738579  KP738580  KP738581 | KP738539  KP738540  KP738541 | KP738499  KP738500  KP738501 | PP280441  PP280442  OL191323 | PP259888  PP259889  PP259890 | PP259683  -  - |
| *Schistura fusinotata* | Laos | Xekong | Mekong | A5065 | PP279991 | PP315830 | PP280188 | PP280430 | PP259880 | - |
| *Schistura implicata* | Vietnam | Lam Dong | No details | GenBank | MG238289 | MG237995 | MG238406 | - | - | - |
| *Schistura incerta* | China | No details | No details | GenBank | MK361215 | KP695623 | KP695078 | - | KP695739 | KP694654 |
| *Schistura* sp. *Lam* | Vietnam | Nghe An | Lam | A3198  A3199 | PP279962  PP279963 | PP315798  PP315799 | PP280169  PP280170 | PP280389  PP280390 | PP259842  PP259843 | PP259667  - |
| *Schistura irregularis* | Laos | Houaphan | Mekong | CMK25919_1  CMK25919_2 | PP279866  PP279867 | PP315701  PP315702 | PP280084  PP280085 | PP280279  PP280280 | -  - | PP259635  - |
| *Schistura kaysonei* | Laos | Bolikhamsay | Mekong | GenBank | NC_031580 | - | - | - | - | - |
| *Schistura klydonion* | Laos | Champasak | Mekong | CMK23320_1  CMK23320_2 | PP279858  PP279859 | PP315693  PP315694 | PP280080  PP280081 | PP280271  PP280272 | PP259980  PP259981 | PP259709  - |
| *Schistura kongphengi* | Vietnam | Thua Thien Hue | Mekong | A2722  A3321 | PP279945  PP279969 | PP315782  PP315805 | PP280155  PP280173 | PP280372  PP280399 | PP259827  PP259851 | -  - |
| *Schistura laterimaculata* | Thailand | Petchabun | Mekong | A6848  A6849 | PP280023  PP280024 | PP315856  PP315857 | PP280208  PP280209 | PP280465  PP280466 | PP259922  PP259923 | PP259689  - |
| *Schistura latidens* | China | Yunnan | Mekong | GenBank | MG238266 | MG237973 | MG238383 | - | MG238081 | - |
| *Schistura latifasciata* | China | Yunnan | Mekong | GenBank | MG238268 | MG237975 | MG238385 | - | MG238083 | - |
| *Schistura macrocephala* | China | Yunnan | Mekong | GenBank | MG238274 | MG237981 | MG238391 | - | MG238088 | - |
| *Schistura macrotaenia* | China | Yunnan | Mekong | GenBank | JN837655 | JN837667 | - | - | - | - |
| *Schistura magnifluvis* | China | Yunnan | Mekong | GenBank | JN837654 | MG237967 | MG238355 | - | MG238075 | - |
| *Schistura moeiensis* | Thailand | Tak | Salween | A4965  A4966  A11033 | PP279988  PP279989  PP279883 | PP315826  PP315827  PP315718 | PP280185  PP280186  PP280094 | PP280426  PP280427  PP280296 | PP259876  PP259877  PP259752 | -  -  - |
| *Schistura nicholsi* | China | Yunnan | Mekong | GenBank | DQ105202 | - | - | - | - | - |
| *Schistura notasileum* | China | Yunnan | Mekong | GenBank | OQ945050 | OQ973300 | OQ973298 | - | OQ973302 | - |
| *Schistura porthos* | China | Yunnan | Mekong | GenBank | MG238282 | MG237988 | MG238399 | - | - | - |
| *Schistura reidi* | Thailand | Mae Hong Son | Salween | A781  A817 | PP280032  PP280036 | PP315865  PP315871 | PP280217  PP280220 | PP280488  PP280494 | PP259950  - | -  - |
| *Schistura rikiki* | Laos | Xekong | Mekong | A5066  A5067 | PP279992  PP279993 | PP315831  PP315832 | PP280189  PP280190 | PP280431  PP280432 | PP259881  - | PP259682  - |
| *Schistura sertata* | Laos | Luang Prabang | Mekong | A2531  A2531 | PP279936  PP279937 | PP315770  PP315771 | PP280147  PP280148 | PP280361  PP280362 | PP259815  PP259816 | -  - |
| *Schistura sexcauda* | Thailand | Chiang Mai | Chao Phraya | A755 | PP280029 | PP315862 | PP280214 | PP280485 | PP259947 | - |
| *Schistura similis* | Thailand | Tak | Salween | A5004 | PP279990 | PP315828 | PP280187 | PP280428 | PP259878 | - |
| *Schistura sokolovi* | Vietnam | Gia Lei | Song Ba | A3384 | PP279970 | PP315806 | PP280174 | PP280400 | PP259852 | PP259673 |
| *Schistura susannae* | Vietnam | Da Nang | Mong Mo | GenBank | MG238288 | MG237994 | MG238405 | - | - | - |
| *Schistura thanho* | Vietnam | Bien Dinh | Vinh Thanh | A3310 | PP279968 | PP315804 | - | PP280398 | - | - |
| *Schistura waltoni* | Thailand | Chiang Mai | Chao Phraya | A731  A732 | PP280027  PP280028 | PP315860  PP315861 | PP280212  PP280213 | PP280477  PP280478 | PP259934  PP259935 | -  - |
| *Schistura xhatensis* | Laos | Houaphan | Mekong | CMK25920_1  CMK25920_2 | PP279868  PP279869 | PP315703  PP315704 | PP280086  PP280087 | PP280281  PP280282 | PP259736  PP259737 | PP259636  - |
| *Schistura yersini* | Vietnam | Lam Dong | Da Dung | A3206 | PP279964 | PP315800 | PP280171 | PP280391 | PP259844 | PP259668 |
| *Sectoria atriceps* | Thailand | Nan | Chao Phraya | A845  A846  A847  A848  A849 | PP280037  PP280038  PP280039  PP280040  PP280041 | PP315872  PP315873  PP315874  PP315875  PP315876 | PP280221  PP280222  PP280223  PP280224  PP280225 | PP280500  PP280501  PP280502  PP280503  PP280504 | PP259957  PP259960  PP259961  PP259962  PP259963 | -  -  -  -  PP259698 |
| *Sectoria heterognathos* | Laos | Louang Namtha | Mekong | CMK25980 | PP279872 | PP315705 | PP280089 | PP280283 | PP259740 | - |
| *Speonectes tiomanensis* | Malaysia | Pahang | Tioman Island | A1848  A1849 | PP279916  PP279917 | PP315750  PP315751 | PP280128  PP280129 | PP280336  PP280337 | PP259791  PP259792 | -  - |
| *Tuberoschistura baenzingeri* | Thailand | Surat Thani | Tapi | A2340  A4485 | -  - | PP315765  PP315822 | PP280143  - | PP280354  PP280418 | PP259807  PP259866 | PP259657  - |
| *Tuberoschistura cambodgiensis* | Cambodia | Phnom Penh | Mekong | A7949  A7950 | -  - | PP315866  PP315867 | PP280218  PP280219 | PP280489  PP280490 | PP259951  PP259952 | PP259696  - |
|  |  |  |  |  |  |  |  |  |  |  |
| SUNDAIC CLADE |  |  |  |  |  |  |  |  |  |  |
| *Nemacheilus binotatus* | Thailand | Chiang Mai | Chao Phraya | A6926  A6927 | KP738586  KP738587 | KP738546  KP738547 | KP738506  PP280210 | OL191346  PP280469 | PP259926  PP259927 | PP259690  - |
| *Nemacheilus cacao* | Laos | Bolikhamsay | Mekong | ZRC 62554  ZRC 62553 | ON720269  ON720270 | ON720271  ON720272 | PP280244  PP280245 | PP280527  PP280528 | PP259983  - | -  - |
| *Nemacheilus* cf. *tuberigum* | Indonesia | Aceh | Alas | A4536  A4537 | PP279986  PP279987 | PP315823  PP315824 | -  - | PP280419  PP280420 | PP259867  PP259868 | PP259680  - |
| *Nemacheilus masyae* | Thailand  Cambodia | Surat Thani  Siem Reap | Tapi  Mekong | A1421  A1422  A5450 | MW512997  MW512998  PP280010 | MW513120  MW513121  PP315847 | PP280109  PP280110  PP280199 | PP280316  PP280317  PP280453 | PP259771  PP259772  PP259901 | PP259644  -  - |
| *Nemacheilus ornatus* | Thailand | Surat Thani | Tapi | A1402  A1403 | MW513023  MW513024 | MW513146  MW513146 | PP280107  PP280108 | PP280314  PP280315 | PP259769  PP259770 | PP259643  - |
| *Nemacheilus pallidus* | Thailand | Nan | Chao Phraya | A1394 | MW513029 | MW513151 | PP280106 | PP280313 | PP259768 | - |
| *Nemacheilus platyceps* | Thailand | Chanthaburi | Mekong | A856  A857 | MW513045  MW513046 | MW513170  MW513171 | PP280226  PP280227 | PP280505  PP280506 | PP259965  PP259966 | -  - |
| *Nemacheilus saravacensis* | Malaysia | Sarawak | Sabang | A1632 | MW513056 | MW513181 | PP280114 | PP280321 | PP259775 | - |
| *Nemacheilus selangoricus* | Thailand | Nakhon Si Thammarat | Pak Paying | A1437 | MW513062 | MW513186 | PP280111 | PP280318 | - | PP259645 |
| *Nemacheilus spiniferus* | Malaysia | Sarawak | Engkaban | A1640 | MW513079 | MW513203 | - | PP280322 | - | PP259647 |
|  |  |  |  |  |  |  |  |  |  |  |
| BURMESE CLADE |  |  |  |  |  |  |  |  |  |  |
| *Aborichthys kempi* | Myanmar | Kachin | Irrawaddy | CMK25538_1 CMK25538_2 | PP280061  PP280062 | PP315697  PP315698 | -  - | PP280275  PP280276 | PP259732  PP259733 | PP259633  - |
| *Aborichthys* sp. | India | West Bengal | Brahma-putra | A3972  A3973 | PP279977  PP279978 | PP315814  PP315815 | PP280177  PP280178 | PP280407  PP280408 | PP259857  - | -  - |
| *Acanthocobitis* cf. *pavonacea* | Ornamental fish trade | | | A1863  A1864 | MK608119 MK608120 | EF056379 MK608146 | MK608242 MK608243 | PP280338  PP280339 | PP259794  PP259795 | -  - |
| *Paracanthocobitis epimekes* | Thailand  Myanmar | Phang Nga  Tanintharyi | Takua Pa  Tenasserim | A2460  CMK 24940 | MK608038  MK608104 | MK608151  MK608213 | MK608248  MK608307 | PP280357  PP280247 | PP259811  PP259730 | -  - |
| *Paracanthocobitis linypha* | Myanmar | no details |  | A2562  A2563 | MK608044 MK608045 | MK608157 MK608158 | MK608254 MK608255 | PP280365  PP280366 | PP259821  - | -  - |
| *Paracanthocobitis mackenziei* | Ornamental  Nepal | fish trade  Koshi | Ganges | A82  A83  A3437 | EF508598 MK608121  MK608113 | EF056383 MK608127  MK608162 | MK608221 MK608222  MK608259 | PP280495  PP280496  PP280401 | -  -  PP259853 | -  -  - |
| *Paracanthocobitis mandalayensis* | Thailand | Tak | Salween | A11558 | PP279890 | PP315726 | - | PP280304 | PP259759 | - |
| *Paracanthocobitis phuketensis* | Myanmar  Thailand  Thailand  Thailand  Thailand | Tanintharyi  Phatthalung  Trang  Phang Nga  Phang Nga | Tenasserim  Phaniat  Palian  Tam Nang  Takua Pa | CMK 28791  A5190  A5175  A9713  A2466 | MK608107  MK608075  MK608071  MK608099  MK608042 | MK608216  MK608186  MK608182  MK608208  MK608155 | MK608310  MK608281  MK608277  MK608302  MK608252 | PP280246  PP280434  PP280433  PP280526  PP280358 | -  PP259882  -  -  PP259812 | -  -  -  -  - |
| *Paracanthocobitis pictilis* | Ornamental | fish trade |  | A6940  A6941  A10938 | KP738589  KP738590  PP279882 | KP738549  KP738550  PP315715 | KP738509  KP738510  - | OL191347  PP280471  PP280293 | PP259929  -  PP259749 | PP259692  -  - |
| *Paracanthocobitis* sp. Irrawaddy | Myanmar  Myanmar | Magway  Ayeyerwady | Irrawaddy  Irrawaddy | A5774  A6621  A6573 | MK608078  MK608089  MK608086 | MK608189  MK608198  MK608196 | MK608284  MK608293  MK608291 | PP280456  PP280459  PP280458 | PP259905  -  PP259912 | -  -  - |
| *Paracanthocobitis* sp. Rakhine | Myanmar | Rakhine | no details | A5331  A5332 | KP738582 KP738583 | KP738542 KP738543 | KP738502 KP738503 | OL191324  PP280443 | PP259891  PP259892 | -  - |
| *Paracanthocobitis* sp. Sittaung | Myanmar | Mon | Sittaung | A4102 | MK608051 | MK608164 | MK608261 | PP280411 | - | - |
| *Paracanthocobitis zonalternans* | Thailand | Tak | Salween | A4942 | MK608065 | MK608176 | MK608271 | PP280425 | - | - |
| *Schistura* cf. *kohchangensis* | Thailand | Tak | Salween | A11046 | PP279884 | PP315719 | PP280095 | PP280297 | PP259753 | - |
| *Schistura kohchangensis* | Thailand | Chanthaburi | Mekong | A1822  A1823 | PP279912  PP279913 | PP315746  PP315747 | PP280124  PP280125 | PP280332  PP280333 | PP259787  PP259788 | -  - |
| *Schistura savona* | Ornamental fish trade | | | A7530  A7532 | KP738598  KP738599 | KP738558  KP738559 | KP738518  KP738519 | PP280482  OL191354 | PP259939  PP259940 | PP259695  - |
|  |  |  |  |  |  |  |  |  |  |  |
| SOUTHERN CLADE | |  |  |  |  |  |  |  |  |  |
| *Afronemacheilus abyssinicus* | Ethiopia | Amhara | Nile | A11680 | PP279891 | PP315727 | PP280101 | PP280305 | PP259760 | - |
| *Mesonoemacheilus guentheri* | India | Ornamental fish trade | | A2630  A2631  A6935 | PP279943  PP279944 | PP315780  PP315781 | PP280153  PP280154 | PP280370  PP280371  PP280470 | PP259825  PP259826  PP259928 | -  -  PP259691 |
| *Mesonoemacheilus herrei* | India | Tamil Nadu | Periyar | A5526  A5527 | PP280011  PP280012 | PP315848  PP315849 | PP280200  PP280201 | PP280454  PP280455 | PP259902  PP259903 | -  - |
| *Mesonoemacheilus pambarensis* | India | Tamil Nadu | Pambar | GenBank | MF680101 | - | - | - | - | - |
| *Mesonoemacheilus petrubanarescui* | India | Karnataka | Saklshpur | A9197 | PP280055 | PP315890 | PP280241 | PP280520 | PP259979 | - |
| *Mesonoemacheilus tambaraparniensis* | India | Tamil Nadu | Tamiraparani | GenBank | MF680096 | - | - | - | - | - |
| *Mesonoemacheilus triangularis* | India | Ornamental fish trade | | A944  A945 | PP280058  PP280059 | PP315891  PP315892 | PP280242  PP280243 | PP280521  PP280522 | PP259984  PP259985 | -  - |
| *Mustura bella* | Laos | Louang Namtha | Mekong | CMK 26052 | OL191242 | OL191491 | OL345558 | OL191373 | PP259743 | - |
| *Mustura celata* | Myanmar | Kachin | Irrawaddy | CMK25620_1  CMK25620_2 | PP279863  PP279864 | PP315699  PP315700 | PP280082  PP280083 | PP280277  PP280278 | PP259734  PP259735 | PP259634  - |
| *Mustura geisleri* | Thailand | Chiang Mai | Chao Phraya | A1238  A1239 | OL191158  OL191159 | OL191407  OL191408 | OL345477  OL345478 | OL191270  OL191271 | PP259764  PP259765 | -  - |
| *Mustura isostigma* | Laos | Khammouane | Mekong | A1307 | PP279899 | - | - | - | - | - |
| *Mustura maepaiensis* | Thailand | Mae Hong Son | Salween | A799  A804  A805 | PP280033  PP280034  PP280035 | PP315868  PP315869  PP315870 | -  -  - | PP280491  PP280492  PP280493 | -  -  - | -  -  - |
| *Mustura shanensis* | Myanmar | Shan | Salween | A6649  A6773  A6774 | OL191213  OL191220  OL191221 | OL191462  OL191469  OL191470 | OL345529  OL345536  OL345537 | OL191333  OL191340  OL191341 | PP259913  -  PP259920 | -  PP259688  - |
| *Mustura* sp. | Myanmar | Magway | Irrawaddy | A6712  A6713 | PP280019  PP280020 | PP315852  PP315853 | PP280204  PP280205 | PP280461  PP280462 | PP259917  - | -  - |
| *Mustura* sp. Salween | Thailand | Tak | Salween | A1776 | PP279907 | - | - | - | - | - |
| *‘Nemacheilus'*  *corica* | India | Ornamental fish trade | | A6945  A6948  A6953 | KP738592  KP738593  KP738594 | KP738552  KP738553  KP738554 | KP738512  KP738513  KP738514 | PP280472  PP280473  PP280475 | PP259930  PP259931  PP259932 | PP259694  -  - |
| *Nemachilichthys ruppelli* | India | Ornamental fish trade | | A4341  A4345 | KP738573  KP738574 | KP738533  KP738534 | KP738493  KP738494 | OL191311  PP280417 | PP259864  PP259865 | PP259678  PP259679 |
| *Neonoemacheilus labeosus* | Thailand | Tak | Salween | A1774  A1775 | PP279905  PP279906 | PP315740  PP315741 | PP280118  PP280119 | PP280326  PP280327 | PP259780  PP259781 | -  - |
| *Neonoemacheilus peguensis* | Myanmar | Bago | Sittaung | A877 | PP280046 | PP315881 | PP280232 | PP280511 | PP259971 | - |
| *Neonoemacheilus* sp. Irrawaddy | Myanmar | Magway | Irrawaddy | A6287 | PP280017 | PP315850 | PP280202 | PP280457 | PP259909 | - |
| *Neonoemacheilus* sp. | Myanmar | Magway | Irrawaddy | A6664  A6734  A6735 | PP280018  PP280021  PP280022 | PP315851  PP315854  PP315855 | PP280203  PP280206  PP280207 | PP280460  PP280463  PP280464 | PP259914  -  - | -  -  - |
| *Oxynoemacheilus angorae* | No details | | | GenBank | NC_031548 | - | - | - | - | - |
| *Oxynoemacheilus brandtii* | Armenia | Lori | Kura | A3901  A3902 | PP279975  PP279976 | PP315812  PP315813 | -  - | PP280405  PP280406 | -  - | -  - |
| *Oxynoemacheilus bureschi* | Bulgaria | Blagoevgrad | Struma | A12147  A12148 | PP279895  PP279896 | PP315731  PP315732 | PP280102  PP280103 | PP280309  PP280310 | PP259991  PP259992 | PP259641  - |
| *Oxynoemacheilus cilicic* | Turkey | Adana | Seyhan | A2758 | PP279947 | PP315784 | PP280157 | PP280374 | PP259828 | - |
| *Oxynoemacheilus chomanicus* | Iran | Kurdistan | Tigris | GenBank | KT715806 | - | - | - | - | - |
| *Oxynoemacheilus euphraticus* | Turkey | Mus | Euphrat | A1003 | PP279876 | PP315708 | PP280090 | PP280286 | - | - |
| *Oxynoemacheilus frenatus* | Turkey | Diyarbakir | Tigris | A2102  A2103 | PP279926  PP279927 | PP315759  PP315760 | PP280137  PP280138 | PP280348  PP280349 | -  - | -  - |
| *Oxynoemacheilus galilaeus* | Syria | Dara | Jordan | A3890  A3891 | PP279973  PP279974 | PP315810  PP315811 | -  PP280176 | -  - | -  - | -  - |
| *Oxynoemacheilus gyndes* | Iraq | Sulaimaniyah | Tigris | GenBank | MH842968 | MH843091 | - | - | - | - |
| *Oxynoemacheilus hanae* | Iraq | Sulaimaniyah | Tigris | GenBank | MH842969 | MH843092 | - | - | - | - |
| *Oxynoemacheilus* veyselorum | Turkey | Erzurum | Arax | A1045 | PP279877 | PP315709 | - | PP280287 | - | - |
| *Oxynoemacheilus insignis* | Israel | West bank | Jordan | A1961  A1962 | PP279920  PP279921 | PP315754  PP315755 | PP280131  PP280132 | PP280342  PP280343 | PP259796  PP259797 | PP259654  - |
| *Oxynoemacheilus kaynaki* 1 | Turkey | Elazig | Euphrat | A1000 | PP279874 | PP315706 | - | PP280284 | - | - |
| *Oxynoemacheilus kaynaki* 2 | Turkey | Elazig | Euphrat | A1002 | PP279875 | PP315707 | - | PP280285 | - | - |
| *Oxynoemacheilus kurdistanicus* | Iran | Kurdistan | Tigris | GenBank | KU180210 | - | - | - | - | - |
| *Oxynoemacheilus merga* | Russia | Dagestan | Rubas | A1109  A1110 | PP279885  PP279886 | PP315720  PP315721 | PP280096  - | PP280298  PP280299 | -  - | PP259637  - |
| *Oxynoemacheilus persa* | Iran | Fars | Kor | A695  A697 | PP280025  PP280026 | PP315858  PP315859 | PP280211  - | PP280474  PP280476 | PP259933  - | -  - |
| *Oxynoemacheilus phasicus* | Georgia | Imereti | Rioni | A2057 | PP279924 | - | PP280135 | PP280346 | PP259800 | - |
| *Oxynoemacheilus pindus* | Albania | Gjirokaster | Vjosa | A12149  A12150 | PP279897  PP279898 | PP315733  PP315734 | PP280104  PP280105 | PP280311  PP280312 | PP259993  PP259994 | PP259642  - |
| *Oxynoemacheilus* sp. Turkey | Turkey | No details | No details | GenBank | EU015983 | - | - | - | - | - |
| *Oxynoemacheilus tongiorgii* | Iran | Fars | Kor | A2757 | PP279946 | PP315783 | PP280156 | PP280373 | - | - |
| *Oxynoemacheilus zagrosensis* | Iran | Kurdistan | Tigris | GenBank | KU180203 | - | - | - | - | - |
| *Oxynoemacheilus zarzianus* | Iraq | Al-Sulaimaniyah | Tigris | GenBank | KY849795 | - | - | - | - | - |
| *Paracobitis atrakensis* | Iran | Khorasane - Shomali | Atrak | GenBank | MG229862 | - | - | - | - | - |
| *Paracobitis hircanica* | Iran | Golestan | Gorgan Roud | A2186  A2187  A2188 | PP279928  PP279929  PP279930 | PP315761  PP315762  PP315763 | PP280139  PP280140  PP280141 | PP280350  PP280351  PP280352 | PP259802  PP259803  PP259804 | PP259656  -  - |
| *Paracobitis malapterura* | Iran | Qom | Emamzadeh Abdollah | GenBank | MG229879 | - | - | - | - | - |
| *Paracobitis molavii* | Iran | West Azerbaijan | Tigris | GenBank | MG229860 | - | - | - | - | - |
| *Paracobitis persa* | Iran | Fars | Kor | GenBank | MG229866 | - | - | - | - | - |
| *Paracobitis rhadinaea* | Iran | Sistan and Baluchistan | Sistan | GenBank | MG229870 | - | - | - | - | - |
| *Paraschistura cristata* | Iran | Khorazan Razavi | Hari | A5262  A5263  A5264  A5265  A5266 | PP279995  PP279996  PP279997  PP279998  PP279999 | PP315833  PP315834  PP315835  PP315836  PP315837 | -  -  -  -  - | PP280436  PP280437  PP280438  PP280439  PP280440 | PP259883  PP259884  PP259885  PP259886  PP259887 | -  -  -  -  - |
| *Paraschistura montana* | India | No details | Ganges | GenBank | FJ711438 | - | - | - | - | - |
| *Petruichthys brevis* | Myanmar | Shan | Salween | A4184  A4185  A6503  A6739 | KP738571  KP738572  OL191210  OL191219 | KP738531  KP738532  OL191459  OL191468 | KP738491  KP738492  OL345526  OL345535 | OL191307  OL191308  OL191330  OL191339 | PP259859  PP259860  PP259910  PP259919 | -  -  -  - |
| *Physoschistura brunneana* | Myanmar | Shan | Salween | A578 | OL191138 | OL191386 | OL345460 | OL191249 | PP259906 | PP259687 |
| *Physoschistura* cf. *rivulicola* | Myanmar | Shan | Salween | A6806 | OL191223 | OL191472 | OL345539 | OL191343 | PP259921 | - |
| *Physoschistura* cf. *shuangjiangensis* | China | Yunnan | Mekong | A2999 | OL191173 | OL191423 | OL345492 | OL191288 | - | - |
| *Physoschistura mango* | Myanmar | Shan | Salween | A2253 | PP279931 | PP315764 | PP280142 | PP280353 | PP259805 | - |
| *Physoschistura pseudobrunneana* | Thailand  Thailand | Phayao  Chiang Rai | Chao Phraya  Mekong | A850  A1356  A1357 | OL191155  OL191163  OL191164 | OL191403  OL191412  OL191413 | OL345474  OL345482  OL345483 | OL191266  OL191275  OL191276 | PP259964  PP259766  PP259767 | -  -  - |
| *Physoschistura rivulicola* | Myanmar | Shan | Salween | A6670 | OL191214 | OL191463 | OL345530 | OL191334 | PP259915 | - |
| *Physoschistura shuangjiangensis* | China | Yunnan | Mekong | GenBank | MG238284 | MG237990 | MG238401 | - | - | - |
| *Physoschistura* sp. | Myanmar | Shan | Salween | A2256  A7545  A7546 | OL191165  KP738600  KP738601 | OL191415  KP738560  KP738561 | OL345484  KP738520  KP738521 | OL191280  OL191355  OL191356 | PP259806  PP259943  PP259944 | -  -  - |
| *Pteronemacheilus lucidorsum* | Myanmar | Shan | Irrawaddy | A6695  A8465  A8466 | OL191215  KP738606  KP738607 | OL191464  KP738566  KP738567 | OL345531  KP738526  KP738527 | OL191335  OL191360  OL191361 | PP259916  PP259958  PP259959 | -  -  - |
| *Pteronemacheilus meridionalis* | China  Laos | Yunnan  Louang Namtha | Salween  Mekong | A3122  CMK 26015  CMK 25928 | PP279958  PP279873  PP279870 | PP315795  PP315676  PP315675 | -  -  - | PP280385  PP280250  PP280248 | -  -  - | -  -  - |
| *Pteronemacheilus* sp. | Myanmar | Shan | Irrawaddy | A5851  A5852 | OL191208  OL191209 | OL191457  OL191458 | OL345524  OL345525 | OL191328  OL191329 | PP259907  PP259908 | -  - |
| *Sasanidus kermanshahensis* | Iran | Kermanshah | Tigris | A5261 | PP279994 | - | - | PP280435 | - | - |
| *Schistura albirostris* | China | Yunnan | Irrawaddy | GenBank | MG238242 | - | MG238356 | - | - | - |
| *Schistura ataranensis* | Myanmar  Thailand | Ornamental  Kanchanaburi | fish trade  Ataran | A2560  A5062  A11005 | MK886975  MK886998  MK887031 | PP315774  PP315829  PP315716 | MK886884  MK886907  MK886939 | PP280364  PP280429  PP280294 | PP259820  PP259879  PP259750 | -  -  - |
| *Schistura aurantiaca* | Myanmar  Thailand  Thailand | Mon  Tak  Tak | Ataran  Mae Klong  Salween | A954  A9580  A9584  A11010 | MK886950  MK887018  MK887019  MK887032 | PP315893  OL191503  PP315894  PP315717 | MK886861  MK886926  MK886927  MK886940 | PP280523  OL191385  PP280524  PP280295 | PP259986  PP259987  PP259988  PP259751 | -  PP259710  -  - |
| *Schistura balteata* | Myanmar | Ornamental fish trade | | A2554  A2555 | MK886971  MK886972 | OL191502  PP315773 | MK886880  MK886881 | OL191384  PP280363 | PP259819  - | -  - |
| *Schistura beavani* | India | No detail | Ganges | GenBank | GQ478448 | - | - | - | - | - |
| *Schistura callidora* | Myanmar | Shan state | Irrawaddy | A3909  A3910 | OL191189  OL191190 | OL191438  OL191439 | OL345507  OL345508 | OL191303  OL191304 | -  - | -  - |
| *Schistura* cf. *nilgiriensis* | India | Karnataka | Netravanthi | A9199 | PP280057 | - | - | - | - | - |
| *Schistura* cf.  *poculi* 1 | Thailand | Tak | Mae Klong | A4202  A4203 | OL191193  OL191194 | OL191442  OL191443 | OL345511  OL345512 | OL191309  OL191310 | -  - | PP259675  - |
| *Schistura* cf.  *poculi* 2 | China | Yunnan | Salween | A2914 | OL191172 | OL191422 | OL345491 | OL191287 | PP259829 | - |
| *Schistura* cf. *scaturigina* | India | West Bengal | Rydak I River | A3925  A3926 | OL191191  OL191192 | OL191440  OL191441 | OL345509  OL345510 | OL191305  OL191306 | PP259856  - | -  - |
| *Schistura* cf. *sijuensis* | India | Ornamental fish trade | | A3698  A3699  A11327 | OL191184  OL191185  OL191248 | OL191434  OL191435  OL191499 | OL345503  OL345504  OL345566 | OL191299  OL191300  OL191381 | -  -  - | -  -  - |
| *Schistura* cf. *vinciguerrae* | Myanmar  Myanmar | Rakhine  Magway | Irrawaddy  Irrawaddy | A5557  A6564  A6736 | OL191205  OL191211  OL191216 | OL191454  OL191460  OL191465 | OL345521  OL345527 OL345532 | OL191325  OL191331  OL191336 | PP259904  PP259911  PP259918 | -  -  - |
| *Schistura cincticauda* | Thailand | Tak | Salween | A8312  A8313 | MK887016  MK887017 | -  - | MK886924  MK886925 | PP280497  PP280498 | PP259953  PP259954 | -  - |
| *Schistura conirostris* | China | Yunnan | Mekong | GenBank | MG238247 | MG237953 | MG238362 | - | MG238061 | - |
| *Schistura crabro* | Laos | Bolikhamsai | Mekong | CMK 24559 | OL191231 | OL191480 | OL345547 | OL191362 | PP259982 | - |
| *Schistura crocotula* | Thailand | Prachuap Khiri Khan | Bang Saphan | A9591  A10513 | MK887021  MK887024 | PP315895  PP315713 | MK886928  MK886931 | PP280525  PP280290 | PP259989  PP259746 | -  - |
| *Schistura denisoni* | India | Tamil Nadu | Vaigai | A5531  A5532 | PP280015  PP280016 | -  - | -  - | -  - | -  - | -  - |
| *Schistura devdedi* | Nepal  India | No detail  Ornamental | Ganges  fish trade | A3445  A7541  A7542 | PP279972  KP738608  KP738609 | PP315808  KP738568  KP738569 | PP280175  KP738528  KP738529 | PP280403  PP280483  PP280484 | -  PP259941  PP259942 | -  -  - |
| *Schistura disparizona* | China | Yunnan | Salween | GenBank | MG238252 | MG238368 | MG237958 | - | MG238065 | - |
| *Schistura hartli* | Thailand | Surat Thani | Tapi | A3690 | MK886978 | PP315809 | MK886887 | PP280404 | PP259855 | - |
| *Schistura hoai* | Laos | Houaphan | Mekong | CMK25918_1 CMK25918_2 | OL191238  OL191239 | OL191487  OL191488 | OL345554  OL345555 | OL191369  OL191370 | -  - | -  - |
| *Schistura hypsiura* | Myanmar | Ornamental fish trade | | A6922  A6925 | KP738584  KP738585 | KP738544  KP738545 | KP738504  KP738505 | PP280467  PP280468 | PP259924  PP259925 | -  - |
| *Schistura indawgyiana* | Myanmar | Kachin | Irrawaddy | CMK 25633 | PP279865 | - | - | - | - | - |
| *Schistura jarutanini* | Thailand | Kanchanaburi | Mae Klong | GenBank | NC_031584 | - | - | - | - | - |
| *Schistura kloetzliae* | Laos | Louang Namtha | Mekong | CMK25994_1 CMK25994_2  GenBank | OL191240  OL191241  MG238237 | OL191489  OL191490  MG237945 | OL345556  OL345557  MG238351 | OL191371  OL191372  - | PP259741  PP259742  - | -  -  - |
| *Schistura kuehnei* | Thailand | Surat Thani | Tapi | A4672  A11261 | MK886991  MK887040 | PP315825  PP315724 | MK886900  PP280099 | PP280422  PP280302 | PP259871  PP259756 |  |
| *Schistura longa* | China | Yunnan | Salween | GenBank | MG238272 | MG237979 | MG238389 | - | MG238086 | - |
| *Schistura mahnerti* | Thailand | Mae Hong Son | Salween | A778  A779 | PP280030  PP280031 | PP315863  PP315864 | PP280215  PP280216 | PP280486  PP280487 | PP259948  PP259949 | -  - |
| *Schistura malaisei* | Myanmar | Kachin | Irrawaddy | CMK25506_1 CMK25506_2 | PP279860  PP279861 | -  PP315696 | -  - | -  - | -  - | -  - |
| *Schistura mukambbikaensis* | India | Karnataka | Netravathi | A9198 | PP280056 | - | - | - | - | - |
| *Schistura myaekanbawensis* | Myanmar | Tanintharyi | Tenasserim | CMK24993 | MK887022 | PP315695 | MK886929 | PP280273 | PP259731 | - |
| *Schistura nilgiriensis* | India | Ornamental fish trade | | A2626 | PP279942 | PP315779 | PP280152 | PP280369 | PP259824 | - |
| *Schistura notostigma* | Sri Lanka | Ornamental fish trade | | A7519  A7520  A7521 | KP738595  KP738596  KP738597 | KP738555  KP738556  KP738557 | KP738515  KP738516  KP738517 | PP280479  PP280480  PP280481 | PP259936  PP259937  PP259938 | -  -  - |
| *Schistura nubigena* | Myanmar | Kachin | Irrawaddy | CMK 25509 | PP279862 | - | - | PP280274 | PP259990 | - |
| *Schistura obliquofascia* | India | No details | No details | GenBank | HM636831 | - | - | - | - | - |
| *Schistura paucicincta* | Thailand | Tak | Salween | A4946  A4947 | OL191203  OL191204 | OL191452  OL191453 | OL345519  OL345520 | OL191321  OL191322 | PP259874  PP259875 | -  - |
| *Schistura polytaenia* | China | Yunnan | Irrawaddy | GenBank | MG238280 | MG237986 | MG238397 | - | MG238092 | - |
| *Schistura pridii* | Thailand | Ornamental fish trade | | A7548  A7549 | KP738602  KP738603 | KP738562  KP738563 | KP738522  KP738523 | OL191357  OL191358 | PP259945  PP259946 | -  - |
| *Schistura reticulofasciata* | India | Assam | Brahmaputra | GenBank | KY379150 | - | - | - | - | - |
| *Schistura robertsi* | Thailand | Phang Nga | Tam Nang | A2424 | MK886959 | OL191501 | MK886869 | OL191383 | PP259810 | - |
| *Schistura rupecula* | Nepal | No detail | Ganges | A3443 | PP279971 | PP315807 | - | PP280402 | PP259854 | - |
| *Schistura semiarmatus* | India | Tamil Nadu | Vaigai | A5529  A5530 | PP280013  PP280014 | -  - | -  - | -  - | -  - | -  - |
| *Schistura sikmaensis* | China | Yunnan | Irrawaddy | GenBank | JF340405 | JF340413 | - | - | - | - |
| *Schistura* sp. *Myanmar* | Myanmar | Ornamental fish trade | | A2569  A2570 | PP279938  PP279939 | PP315775  PP315776 | -  - | -  - | -  - | -  - |
| *Schistura thavonei* | Laos | Louang Namtha | Mekong | CMK 26066 CMK25944_1 | OL191243  OL191244 | OL191492  OL191493 | OL345559  OL345560 | OL191374  OL191375 | PP259744  PP259739 | -  - |
| *Schistura tirapensis* | India | Ornamental fish trade | | A3703  A3704 | OL191187  OL191188 | OL191436  OL191437 | OL345505  OL345506 | OL191301  OL191302 | -  - | -  - |
| *Schistura udomritthiruji* | Thailand | Ranong | Kapoe | A2546  A2547 | OL191168  MK886969 | OL191418  PP315772 | OL345487  PP280149 | OL191283  - | PP259817  PP259818 | PP259661  - |
| *Schistura yingjiangensis* | China | Yunnan | Irrawaddy | GenBank | MG238294 | MG237999 | MG238411 | - | MG238103 | - |
| *Seminemacheilus ispartensis* | Turkey | Isparta | Egidir | A4833  A4834 | KP738577  KP738578 | KP738537  KP738538 | KP738497  KP738498 | PP280423  PP280424 | PP259872  PP259873 | -  - |
| *Turcinoemacheilus ekmekciae* | Turkey | Diyarbakir | Tigris | A2089 | PP279925 | PP315758 | PP280136 | PP280347 | PP259801 | - |
| *Turcinoemacheilus* sp. 1 | Iran | Kurdistan | Choman | GenBank | KT861416 | - | - | - | - | - |
| *Turcinoemacheilus* sp. 2 | Iran | Khuzestan | Karoon | GenBank | GQ338827 | - | - | - | - | - |
|  |  |  |  |  |  |  |  |  |  |  |
| OUTGROUP |  |  |  |  |  |  |  |  |  |  |
| Catostomidae |  |  |  |  |  |  |  |  |  |  |
| *Cycleptus elongatus* |  |  |  | GenBank | NC_031634  14392-15532 | EU409613 | EU409671 | - | EU409639 | EU409767 |
| *Catostomus commersonii* |  |  |  | GenBank | JX488781 | EU409612 | EU409670 | FJ918841 | EU409638 | EU409766 |
| *Hypentelium nigricans* |  |  |  | GenBank | AF454909 | EU711134 | JX470004 | JX190418 | FJ197033 | - |
| Gyrinocheilidae |  |  |  |  |  |  |  |  |  |  |
| *Gyrinocheilus aymonieri* |  |  |  | GenBank | NC_008672 14393-15533 | EU292682 | FJ197122 | - | FJ197071 | EU409791 |
| *Gyrinocheilus pennocki* |  |  |  | GenBank | NC_031544 14395-15535 | FJ650415 | FJ650486 | - | FJ650474 | FJ650461 |
| Botiidae |  |  |  |  |  |  |  |  |  |  |
| *Leptobotia pellegrini* |  |  |  | GenBank | NC_031602 14385-15525 | EU292683 | EU409672 | - | EU409640 | EU409768 |
| *Botia dario* |  |  |  | GenBank | KU517084 | KU517026 | MF681756 | - | EU409641 | - |
| *Yasuhikotakia morleti* |  |  |  | GenBank | NC_031600 14377-15517 | FJ650412 | FJ650483 | - | FJ650471 | FJ650457 |
| *Syncrossus beauforti* |  |  |  | GenBank | NC_031546 14387-15527 | FJ650411 | FJ650482 | - | FJ650470 | FJ650456 |
| Vaillantellidae |  |  |  |  |  |  |  |  |  |  |
| *Vaillantella maassi* |  |  |  | GenBank | NC_008680 14378-15518 | EU711132 | FJ197080 | - | FJ197031 | FJ650469 |
| Cobitidae |  |  |  |  |  |  |  |  |  |  |
| *Pangio oblonga* |  |  |  | GenBank | NC_031592 14386-15526 | EU711141 | FJ197091 | - | FJ197041 | FJ650459 |
| *Canthophrys gongota* |  |  |  | GenBank | NC_031576 14380-15516 | FJ650414 | FJ650485 | - | FJ650473 | FJ650460 |
| *Cobitis takatsuensis* |  |  |  | GenBank | NC_015306 14445-15585 | EU409616 | EU409675 | - | EU409643 | EU409771 |
| *Cobitis lutheri* |  |  |  | GenBank | JN858887 | EF508614 | KM818238 | KM818242 | KM583634 | - |
| *Cobitis taenia* | Germany | Lower Saxonia | Weser | A1860 | EF508508 | EF056334 | MK608315 | OL191279 | PP259793 | PP259653 |
| *Cobitis multifasciata* |  |  |  | GenBank | NC_027166 14376-15516 | EU409615 | EU409674 | - | EU409642 | EU409770 |
| *Cobitis tetralineata* |  |  |  | GenBank | KF661673 | OK661770 | - | OK661564 | - | - |
| Ellopostomatidae |  |  |  |  |  |  |  |  |  |  |
| *Ellopostoma mystax* |  |  |  | GenBank | NC_031642 14377-15517 | FJ650417 | FJ650489 | - | FJ650477 | FJ650464 |
| Balitoridae |  |  |  |  |  |  |  |  |  |  |
| *Homaloptera parclitella* |  |  |  | GenBank | NC_031634 14392-15532 | EU409610 | EU409668 | - | EU409636 | EU409764 |
| Gastromyzonidae |  |  |  |  |  |  |  |  |  |  |
| *Sewellia lineolata* |  |  |  | GenBank | NC_015534 14375-515 | EU409609 | EU409667 | - | EU409635 | EU409763 |
|  |  |  |  |  |  |  |  |  |  |  |
| Cyprinidae |  |  |  |  |  |  |  |  |  |  |
| *Enteromius callipterus* |  |  |  | GenBank | KP712230 | FJ531247 | FJ531365 | - | FJ531345 | FJ531317 |
