# Supplementary material for: Reconstructing the phylogeny and evolutionary history of freshwater fishes (Nemacheilidae) across Eurasia since early Eocene
Source: eLife. 2025 Apr 4;13:RP101080. doi: 10.7554/eLife.101080 (PMC11970906; doi:10.7554/eLife.101080)
Supplement: Supplementary file 2. [file elife-101080-supp2.docx]

**Table S2.**

List of primers used in the present study for amplification and/or sequencing.

| Locus | Primer name | Primer sequence (5’ - 3’) | Reference |
| --- | --- | --- | --- |
| Cyt *b* | Glu-L.Ca14337–14359 | GAA GAA CCA CCG TTG TTA TTC AA | Šlechtová et al., 2006 |
|  | Thr-H.Ca15568–15548 | *ACC TCC RAT CTY CGG ATT ACA* | Šlechtová et al., 2006 |
|  | CB-L.Ca14975–14994 | CAC GAR ACR GGR TCN AAY AA | Šlechtová et al., 2006 |
|  | CB-H.Ca15057–15035 | *TCT TTR TAT GAG AAR TAN GGG TG* | Šlechtová et al., 2006 |
| IRBP 2 | 101F | TCM TGG ACA AYT ACT GCT CAC C | Chen et al., 2008 |
|  | 109F | AAC TAC TGC TCR CCA GAA AAR C | Chen et al., 2008 |
|  | 1001R | *GGA AAT GCA TAG TTG TCT GCA A* | Chen et al., 2008 |
|  | 1162R | *TGG TGG WCT TYA GGC ACT TGT* | Chen et al., 2008 |
| RAG 1 | RAG-1F | AGC TGT AGT CAG TAY CAC AAR ATG | Grande et al., 2004 |
|  | RAG-RV1 | *TCC TGR AAG ATY TTG TAG AA* | Šlechtová et al., 2007 |
| MYH 6 | myh6-F507 | GGA GAA TCA RTC KGT GCT CAT CA | Li et al., 2007 |
|  | myh6-R1322 | *CTC ACC ACC ATC CAG TTG AAC AT* | Li et al., 2007 |
| RH 1 | RH-1F | CAT ACG AAT ATC CCC AGT ACT ACC | Liu et al., 2012 |
|  | RH-28F | TAC GTG CCT ATG TCC AAY GC | Chen et al. 2008 |
|  | RH-139F | CNT ATG AAT AYC CTC AGT ACT ACC | Chen et al. 2003 |
|  | RH-233F | ATA TGC CTG CCT GGC YGC TTA C | Chen et al. 2008 |
|  | RH-1R | *GCT TGT TCA TGC AGA TGT AGA TGC* | Liu et al., 2012 |
|  | RH-1039R | TGC TTG TTC ATG CAG ATG TAG A | Chen et al. 2003 |
| EGR 3 | E3-161F | AAT ATC ATG GAC YTG GGN ATG G | Chen et al. 2008 |
|  | E3-1136R | GGY TTC TTG TCC TTC TGT TTS AG | Chen et al. 2008 |

Chen, W.-J., Miya, M., Saitoh, K., Mayden, R.L., 2008. Phylogenetic utility of two existing and four novel nuclear gene loci in reconstructing Tree of Life of ray-finned fishes: The order Cypriniformes (Ostariophysi) as a case study. Gene 423, 125–134.

Grande, T., Laten, H., Lopez, J.A., 2004. Phylogenetic relationships of extant esocid species (Teleostei: Salmoniformes) based on morphological and molecular characters. Copeia 743–757.

Li, C., Orti, G., Zhang, G., Lu, G. 2007. A Practical Approach to Phylogenomics: The Phylogeny of Ray-Finned Fish (Actinopterygii) as a Case Study. BMC Evol. Biol. 7, 44.

Liu, S.-Q.; Mayden, R.L.; Zhang, J.-B.; Yu, D.; Tang, Q.-Y.; Deng, X.; Liu, H.-Z. Phylogenetic relationships of the Cobitoidea (Teleostei: Cypriniformes) inferred from mitochondrial and nuclear genes with analyses of gene evolution. *Gene* **2012**, *508*, 60– 72.

Šlechtová, V., Bohlen, J., Freyhof, J., Ráb, P., 2006. Molecular phylogeny of the Southeast Asian freshwater fish family Botiidae (Teleostei: Cobitoidea) and the origin of polyploidy in their evolution. Mol. Phylogenet. Evol. 39, 529–541.

Šlechtová, V., Bohlen, J. Tan, H.-H. 2007. Families of Cobitoidea (Teleostei; Cypriniformes) as revealed from nuclear genetic data and the position of the mysterious genera Barbucca, Psilorhynchus, Serpenticobitis and Vaillantella. Mol. Phylogenet. Evol. 44, 1358-65.
