# Supplementary material for: Reconstructing the phylogeny and evolutionary history of freshwater fishes (Nemacheilidae) across Eurasia since early Eocene
Source: eLife. 2025 Apr 4;13:RP101080. doi: 10.7554/eLife.101080 (PMC11970906; doi:10.7554/eLife.101080)
Supplement: Supplementary file 3. — Lengths of alignments, numbers of variable (VP), and parsimony informative (PI) positions and models estimated for all partitions. BEAST and MrBayes models were calculated in Partition Finder 2 (PF2, Lanfear et al., 2016) implemented in PhyloSuite v1.2.2 (Zhang et al., 2020) under AICc criterion, with greedy algorithm (Lanfear et al., 2016) and branch lengths linked. For ML trees, the models and partitioning schemes were estimated under BIC with ModelFinder (Kalyaanamoorthy et al., 2017) implemented in IQ tree. The values and models were calculated for both (A) full as well as (B) reduced dataset. Table (C) provides an overview of data attributes for the ingroup dataset only. [file elife-101080-supp3.docx]

**Table S3.**

Alignment attributes and best-fit models. Lengths of alignments, numbers of variable (VP) and parsimony informative (PI) positions and models estimated for all partitions. BEAST and MrBayes models were calculated in Partition Finder 2 (PF2, Lanfear et al., 2016) implemented in PhyloSuite v1.2.2 (Zhang et al., 2020) under AICc criterion, with greedy algorithm (Lanfear et al., 2016) and branch lengths linked. For ML trees the models and partitioning schemes were estimated under BIC by ModelFinder (Kalyaanamoorthy et al. 2017) implemented in IQ tree. The values and models were calculated for both (A) full as well as (B) reduced dataset. Table (C) provides an overview of data attributes for the ingroup dataset only.

| **A: full dataset** |  |  |  |  |  |  |  |
| --- | --- | --- | --- | --- | --- | --- | --- |
| locus |  | EGR3 | IRBP 2 | MYH6 | RAG1 | RH | Cytb |
| length (bp) |  | 876 | 831 | 777 | 950 | 844 | 1122 |
| Variable positions (VP) |  | 341 | 559 | 331 | 503 | 385 | 680 |
| % VP |  | 38.93 | 67.27 | 42.60 | 52.95 | 45.62 | 60.61 |
| Pars. Informative (PI) |  | 268 | 483 | 299 | 453 | 333 | 615 |
| % PI |  | 30.59 | 58.12 | 38.48 | 47.68 | 39.45 | 54.81 |
|  | partition |  |  |  |  |  |  |
| MrBayes models  (PF2, AICc) | gene | GTR+I+G | SYM+I+G | GTR+I+G | SYM+I+G | GTR+I+G | GTR+I+G |
|  | 1st c.p. | GTR+I+G | GTR+I+G | GTR+I+G | GTR+I+G | SYM+I+G | SYM+I+G |
|  | 2nd c.p. | GTR+I+G | GTR+I+G | GTR+I+G | GTR+I+G | GTR+I+G | GTR+I+G |
|  | 3rd c.p. | GTR+I+G | SYM+I+G | SYM+G | SYM+I+G | GTR+G | GTR+G |
| IQ-Tree models (ModelFinder, BIC) | gene | TPM2u+F+I+G4 | TIM2e+I+G4 | TIM2e+I+G4 | TIM2e+I+G4 | TIM2e+I+G4 | TVM+F+I+G4 |
|  | 1st c.p. | TIM2e+I+G4 | TIM3+F+R3 | TN+F+R3 | TIM2e+R3 | TIM2e+R3 | TIM2e+I+G4 |
|  | 2nd c.p. | TIM3e+R2 | TVM+F+G4 | TIM2+F+R3 | TVMe+I+G4 | TPM2+F+R3 | TVM+F+R4 |
|  | 3rd c.p. | GTR+F+G4 | TIM2e+R3 | TIM2e+R4 | TVMe+G4 | TIM2+F+G4 | TIM2+F+ASC+R5 |
| BEAST models  (PF2, AICc) | gene | GTR+I+G+X | GTR+I+G+X | GTR+I+G+X | GTR+I+G+X | GTR+I+G+X | GTR+I+G+X |
|  | 1st c.p. | N/E | N/E | N/E | N/E | N/E | N/E |
|  | 2nd c.p. | N/E | N/E | N/E | N/E | N/E | N/E |
|  | 3rd c.p. | N/E | N/E | N/E | N/E | N/E | N/E |

**Table S3 continuation**

| **B: reduced dataset** | |  |  |  |  |  |  |
| --- | --- | --- | --- | --- | --- | --- | --- |
| locus |  | EGR3 | IRBP 2 | MYH6 | RAG1 | RH | Cytb |
| alignment bp |  | 876 | 831 | 777 | 950 | 844 | 1122 |
| var.pos. |  | 341 | 546 | 327 | 500 | 376 | 681 |
| % V |  | 38.93 | 65.70 | 42.08 | 52.63 | 44.55 | 60.70 |
| Pars. Inf. |  | 268 | 436 | 284 | 427 | 312 | 579 |
| % PI |  | 30.59 | 52.47 | 36.55 | 44.95 | 36.97 | 51.60 |
|  | partition |  |  |  |  |  |  |
| MrBayes models  (PF2, AICc) | gene | GTR+I+G | SYM+I+G | GTR+I+G | SYM+I+G | GTR+I+G | GTR+I+G |
|  | 1st c.p. | GTR+I+G | GTR+I+G | GTR+I+G | SYM+I+G | SYM+I+G | SYM+I+G |
|  | 2nd c.p. | GTR+I+G | GTR+I+G | GTR+I+G | GTR+I+G | GTR+I+G | GTR+I+G |
|  | 3rd c.p. | GTR+I | SYM+I+G | SYM+G | SYM+I+G | GTR+G | GTR+G |
| IQ-Tree models (ModelFinder, BIC) | gene | HKY+F+I+G4 | TIM2e+I+G4 | TN+F+I+G4 | TIM2e+I+G4 | TPM2+F+I+G4 | GTR+F+I+G4 |
|  | 1st c.p. | TIM2+F+I+G4 | TIM3+F+G4 | K2P+I+G4 | TIM2e+I+G4 | TNe+I+G4 | TIM2e+I+G4 |
|  | 2nd c.p. | TIM2+F+I+G4 | TVM+F+G4 | TIM2+F+I+G4 | TIM2e+I+G4 | TVM+F+I+G4 | TVM+F+G4 |
|  | 3rd c.p. | K2P+G4 | TIM2e+G4 | TIM2e+G4 | TVMe+I+G4 | TIM2e+G4 | TIM2+F+ASC+G4 |
| BEAST models (PF2, AICc) | gene | GTR+I+G+X | GTR+I+G+X | GTR+I+G+X | GTR+I+G+X | GTR+I+G+X | GTR+I+G+X |
|  | 1st c.p. | HKY+I+G+X | TRN+I+G+X | TRN+I+G+X | GTR+I+G+X | GTR+I+G+X | GTR+I+G+X |
|  | 2nd c.p. | GTR+I+G+X | GTR+G+X | GTR+I+G+X | GTR+I+G+X | GTR+I+G+X | GTR+I+G+X |
|  | 3rd c.p. | GTR+G+X | GTR+I+G+X | GTR+G+X | GTR+I+G+X | GTR+G+X | GTR+G+X |

| **Table S3 continuation**  **C: full dataset only ingroup** | | |  |  |  |
| --- | --- | --- | --- | --- | --- |
| Locus | length (bp) | VP | % VP | PI | % PI |
| EGR3 | 876 | 286 | 32.65 | 207 | 23.63 |
| IRBP 2 | 831 | 524 | 63.06 | 443 | 53.31 |
| MYH6 | 777 | 313 | 40.28 | 286 | 36.81 |
| RAG1 | 950 | 463 | 48.74 | 425 | 44.74 |
| RH | 844 | 352 | 41.71 | 307 | 36.37 |
| Cytb | 1122 | 668 | 59.54 | 610 | 54.37 |

Lanfear, R., Calcott, B., Ho, S. Y., & Guindon, S. (2012). PartitionFinder: combined selection of partitioning schemes and substitution models for phylogenetic analyses. Molecular biology and evolution, 29(6), 1695-1701.

Lanfear, R., Frandsen, P. B., Wright, A. M., Senfeld, T., Calcott, B. (2016) PartitionFinder 2: new methods for selecting partitioned models of evolution for molecular and morphological phylogenetic analyses. Molecular biology and evolution. DOI: dx.doi.org/10.1093/molbev/msw260

Kalyaanamoorthy S, Minh BQ, Wong TKF, von Haeseler A, Jermiin LS. 2017. ModelFinder: fast model selection for accurate phylogenetic estimates. Nat Methods. 14(6):587–589.

Zhang, D.; Gao, F.; Jakovlić , I.; Zou, H.; Zhang, J.; Li, W.X.; Wang, G.T. PhyloSuite: An integrated and scalable desktop platform for streamlined molecular sequence data management and evolutionary phylogenetics studies. Mol. Ecol. Resour. 2020, 20,348–355
