# Supplementary material for: Reconstructing the phylogeny and evolutionary history of freshwater fishes (Nemacheilidae) across Eurasia since early Eocene
Source: eLife. 2025 Apr 4;13:RP101080. doi: 10.7554/eLife.101080 (PMC11970906; doi:10.7554/eLife.101080)
Supplement: Supplementary file 4. — The last column shows the p-values of the likelihood ratio test. Based on AICc weight (AICc wt), the DEC+J model is recommended as the best fit for our dataset, supported by low p-values indicating the significant influence of the J factor on model likelihood. [file elife-101080-supp4.docx]

**Table S4.**

Comparison of biogeographic models in RASP. The last column shows the p-values of the Likelihood Ratio Test. Based on AICc weight (AICc wt), the DEC+J model is recommended as the best fit for our dataset, supported by low p-values indicating the significant influence of the J factor on model likelihood.

| Model | LnL | numparams | d | e | j | AICc | AICc_wt | LRT p-val |
| --- | --- | --- | --- | --- | --- | --- | --- | --- |
| DEC | -369.3 | 2 | 0.0029 | 0.047 | 0 | 742.6 | 2.0e-26 | 1.2e-27 |
| DEC+J | -309.9 | 3 | 1.0e-12 | 0.017 | 0.013 | 625.8 | 0.45 |  |
| DIVALIKE | -367.2 | 2 | 0.0031 | 0.046 | 0 | 738.5 | 1.5e-25 | 3.3e-26 |
| DIVALIKE+J | -311.2 | 3 | 1.0e-12 | 0.044 | 0.014 | 628.4 | 0.13 |  |
| BAYAREALIKE | -337.9 | 2 | 0.0044 | 0.22 | 0 | 679.8 | 8.7e-13 | 7.8e-14 |
| BAYAREALIKE+J | -310 | 3 | 0.0002 | 0.15 | 0.012 | 626 | 0.42 |  |
